# Prevalence of *Plasmodium falciparum* gametocytaemia in asymptomatic school children before and after treatment with dihydroartemisinin-piperaquine (DP)

**DOI:** 10.1016/j.parepi.2023.e00292

**Published:** 2023-02-17

**Authors:** Bismarck Dinko, Dennis Awuah, Kwadwo Boampong, John A. Larbi, Teun Bousema, Colin J. Sutherland

**Affiliations:** a Department of Infection Biology, London School of Hygiene and Tropical Medicine, London, UK; b Department of Theoretical and Applied Biology, Faculty of Biosciences, College of Science, Kwame Nkrumah University of Science and Technology, Kumasi, Ghana; c Department of Medical Microbiology, Nijmegen Medical Centre, Radboud University, Nijmegen, the Netherlands

**Keywords:** *Plasmodium falciparum*, Gametocytaemia, Asymptomatic infections, School children, ACT, Artemisinin-based combination therapy, DP, dihydroartemisin piperaquine, WHO, world health organization, GNMCP, Ghana national malaria control programme, MDA, mass drug administration, PCR, polymerase chain reaction, WWARN, worldwide antimalarial resistance network, NASBA, nucleic acid sequence-based amplification, RT-qPCR, reverse transcriptase quantitative polymerase chain reaction

## Abstract

**Background:**

Asymptomatic Plasmodium carriers form the majority of malaria-infected individuals in most endemic areas. A proportion of these asymptomatically infected individuals carry gametocytes, the transmissible stages of malaria parasites, that sustain human to mosquito transmission. Few studies examine gametocytaemia in asymptomatic school children who may form an important reservoir for transmission. We assessed the prevalence of gametocytaemia before antimalarial treatment and monitored clearance of gametocytes after treatment in asymptomatic malaria children.

**Methods:**

A total of 274 primary school children were screened for *P. falciparum* parasitaemia by microscopy. One hundred and fifty-five (155) parasite positive children were treated under direct observation with dihydroartemisinin-piperaquine (DP). Gametocyte carriage was determined by microscopy seven days prior to treatment, day 0 before treatment, and on days 7, 14 and 21 post initiation of treatment.

**Results:**

The prevalence of microscopically-detectable gametocytes at screening (day −7) and enrolment (day 0) were 9% (25/274) and 13.6% (21/155) respectively. Following DP treatment, gametocyte carriage dropped to 4% (6/135), 3% (5/135) and 6% (10/151) on days 7, 14 and 21 respectively. Asexual parasites persisted in a minority of treated children, resulting in microscopically detectable parasites on days 7 (9%, 12/135), 14 (4%, 5/135) and 21 (7%, 10/151). Gametocyte carriage was inversely correlated with the age of the participants (*p* = 0.05) and asexual parasite density (*p* = 0.08). In a variate analysis, persistent gametocytaemia 7 or more days after treatment was significantly associated with post-treatment asexual parasitaemia at day 7 (*P* = 0.027) and presence of gametocytes on the day of treatment (*P* < 0.001).

**Conclusions:**

Though DP provides both excellent cure rates for clinical malaria and a long prophylactic half-life, our findings suggest that after treatment of asymptomatic infections, both asexual parasites and gametocytes may persist in a minority of individuals during the first 3 weeks after treatment. This indicates DP may be unsuitable for use in mass drug administration strategies towards malaria elimination in Africa.

## 1 Introduction

*Plasmodium falciparum* is responsible for the most severe form of malaria in humans and kills about 627,000 people yearly, mostly children under 5 years and pregnant women (WHO, 2021). In Ghana, malaria is the number one cause of hospital attendance and admissions, resulting in about 8% mortality for malaria-associated hospitalizations (Ghana national malaria control programme report, 2021). The morbidity and mortality caused by malaria is due to the asexual reproduction of the parasite population in red blood cells (RBCs). The sexual stages of the malaria parasites capable of infecting mosquitoes are called gametocytes. Transmission to the mosquito vector, is achieved only by non-dividing male and female gametocytes, which do not cause disease (Alano, 2007).

The majority of the malaria-infected population in most endemic countries remains asymptomatic, carrying varying levels of parasitaemia without any obvious clinical symptoms that elicit treatment-seeking behavior (Greenwood, 1987; Bousema et al., 2014). Among this asymptomatic population is a large number of gametocyte-positive individuals who may contribute to onward malaria transmission (Slater et al., 2015). Consequently, efforts to reduce malaria transmission in communities may benefit from interventions such as mass drug administration (MDA) that are expected to reduce this asymptomatic reservoir (Johnston et al., 2014).

Human-to-mosquito malaria transmission requires the presence of circulating infectious stage V gametocytes in human peripheral blood (Smalley et al., 1981). Strategies interfering with gametocyte development, infectiousness and availability to biting mosquitoes may reduce malaria transmission. Gametocytes of *P. falciparum* develop as the progeny of sexually-committed schizonts, (Bruce et al., 1990) and *in vitro* differentiate over a period of 8–12 days through 5 morphologically distinct stages within host red blood cells (Alano, 2007). Only mature stage V gametocytes are seen in peripheral circulation *in vivo* while stages I-IV are sequestered, predominantly in the bone marrow (Joice et al., 2014). Whereas these immature gametocytes are susceptible to many anti-malarial drugs, mature gametocytes that are present before treatment commonly persist after treatment (Djimde et al., 2016; Kumar and Zheng, 1990; Lelièvre et al., 2012).

Artemisinin-based combination therapies (ACT) have an incomplete effect on gametocytes (Chen et al., 1994; Bousema et al., 2006; Carrara et al., 2009; White et al., 2009) and field trials have demonstrated that ACT reduces but does not fully prevent post-treatment transmission of *P. falciparum* to *Anopheles gambiae* (Targett et al., 2001; Sutherland et al., 2005; Sawa et al., 2013). The use of ACT such as dihydroartemisinin-piperaquine (DP) in MDA trials has led to reduction, but not cessation, of *P. falciparum* transmission (von Seidlein et al., 2019; Shah et al., 2021), consistent with evidence that asymptomatic *Plasmodium-*infected children in Ghana were not all cleared of parasitaemia by a full three-day therapeutic course of DP (Dinko et al., 2013). As a result, it has been suggested that effective malaria control and ultimately elimination will benefit from, among other things, drugs targeting all stages of the malaria parasite including gametocytes (Djimde et al., 2016).

Most studies assessing gametocytaemia in children have been clinical trials in symptomatic individuals, excluding asymptomatic infections. Longitudinal investigations in asymptomatic school children in Ghana have rarely been conducted. Here we present a short report of a sub-analysis assessing longitudinal carriage of microscopy-detected gametocytaemia in a cohort of asymptomatic school children with confirmed *P. falciparum* infections, treated with DP. These data are from a major study evaluating *P. falciparum* gametocyte immunity in Ghanaian school children (Dinko et al., 2016).

## 2 Materials and methods

### 2.1 Ethics statement

Ethical approval to conduct the study and to use the samples were obtained from the Ghana Health Service Ethics Review Committee in Accra (GHS-ERC-08/7/10) and the London School of Hygiene and Tropical Medicine Ethics Committee (Ref: 2010_5775). In addition, individual informed consent and assent as well as community consent were obtained before the study was conducted. Approval was also obtained from the Ahafo Ano South District Education Directorate and the authorities of the Pokukrom Methodist Primary School since the study involved school children. Given that primary school children represent an age group that require written informed consent to be obtained from their parents before participation, community consent was obtained as part of the community entry to introduce the study and the respective class teachers served as guardians.

### 2.2 Study population and study area

The study participants were primary school children between the ages of 6 and 12 years attending the Methodist School in Pokukrom in the Ahafo Ano South District of the Ashanti Region of Ghana. Pokukrom is within the Ahafo Ano South district and it is an area of high malaria transmission, and with two rainy seasons; a major wet season from April to July and a minor one from September to November (Asante et al., 2011). This particular school was chosen for the study because of the availability of a government-supported school feeding programme, which enhanced the success of our weekly follow-up.

### 2.3 Sampling procedures

Finger-prick blood samples for thick and thin blood smears were obtained weekly from asymptomatic malaria-infected children longitudinally over 5 visits (Visit 1 to visit 5). Asymptomatic malaria-infected children were defined as children who were blood slide positive but without any clinical symptoms and signs of malaria upon clinical examination. The study design, participants, inclusion and exclusion criteria, environment and sampling procedures have been previously described (Dinko et al., 2013; Dinko et al., 2016). Briefly, during the minor rainy season, from October to December 2010, primary school children of the Methodist school in Pokukrom were screened (on day −7, visit 1) for malaria parasites *via* finger-prick blood for microscopy after clinical examination by qualified trained nurses. The inclusion criteria were parasite positivity of any *Plasmodium* species including mixed species infections, any parasitaemia levels without any obvious signs and symptoms of malaria. The main exclusion criteria were malaria symptoms, including recent history of fever, signs and symptoms of chronic and severe diseases and malaria treatment in the last two weeks (Dinko et al., 2016).

### 2.4 Microscopic examination of blood smears

Thick and thin blood smears prepared in the field at each visit were stained with 10% Giemsa the same day upon returning to the laboratory at the Department of Biology, Kwame Nkrumah University of Science and Technology, Kumasi, Ghana. Double parasite readings were independently carried out by counting the number of parasitized erythrocytes per 200 white blood cells for asexual parasites but against 500 white blood cells in the case of sexual parasites. The parasitaemia obtained were expressed as parasites per microliter of blood by assuming there are 8000 leucocytes in one microliter of blood (Greenwood and Armstrong, 1991; Drakeley et al., 2004). The thin films on the slides were used to determine the different malaria species in the study population and these were also examined by two expert microscopists.

### 2.5 Treatment and follow-up

Plasmodium-positive individuals, identified by blood films collected on visit 1, were treated with DP 7 days later (on treatment day 0 (TD0)) at visit 2. by direct observation and supervision by a clinically qualified nurse during school after meals, according to Ghana Government treatment guidelines and recommendations of the approving ethics review committee. Thus, children were enrolled one week after screening, once their test results were known. As it is not national policy to delay treatment of asymptomatic children for a week, in between the screening and enrollment days nurses were at school to monitor children for possible symptoms of malaria such as rising temperatures. Those who showed symptoms of malaria during this time were advised and referred to the health facility. On the day of enrolment (day 0, visit 2) all parasite positive children who were present at school were given treatment and those who were absent were advised to visit the health facility upon their next attendance in school and these were not included in the study. Enrollees were followed up weekly on TD7 (visit 3), TD14 (visit 4) and TD21 (visit 5) with finger-prick blood collections for blood smears on slide, filter paper blood spots and plasma separation. Similarly, nurses were available on all days to monitor the school children during the follow-up to ensure complete compliance of treatment and possible symptoms of malaria. Microscopy and PCR detection of asexual parasite carriage and *Plasmodium* species composition during follow-up has been reported previously (Dinko et al., 2013).

### 2.6 Data analysis

Participant data were recorded manually on an enrollment/case report form capturing demographic data, asexual parasite and gametocyte counts which were then entered into a customized Microsoft Access database. The data were double-entered independently to allow correction of errors and inconsistencies and securely backed up. The outcome measures were gametocytaemia at enrollment, the development and loss of gametocytes during follow-up.

Geometric mean of the independent microscopy readings was used for analyses. Estimations of gametocyte and asexual parasite prevalence on the day of screening (day −7) was based on the full sampled population and subsequent longitudinal analyses considered only those who were parasite-positive on day 0, and thus received treatment. Analyses comparing different days were based on per protocol data. Student's *t*-test was used to determine differences in geometric mean densities of sexual and asexual parasites when normally distributed and in the geometric mean age of gametocyte and non-gametocyte carriers. The Wilcoxon rank-sum test was used to test for association between asexual parasite density and gametocyte prevalence. Age was considered as a continuous variable while asexual parasite and gametocyte prevalence were analysed as binary variables. Asexual parasite and gametocyte densities were considered as continuous variables. Sample size determination was based on previous known asexual parasite prevalence of 65% in a nearby study area and gametocyte prevalence of 25% in a similar endemic area within West Africa. Logistic regression models were used to determine factors associated with gametocyte carriage and persistence over different sampling times. All analyses were performed using Stata software (Stata 14.2, Statacorp, Texas, USA).

## 3 Results

### 3.1 Gametocyte carriage at screening and enrolment

**Fig. 1:**
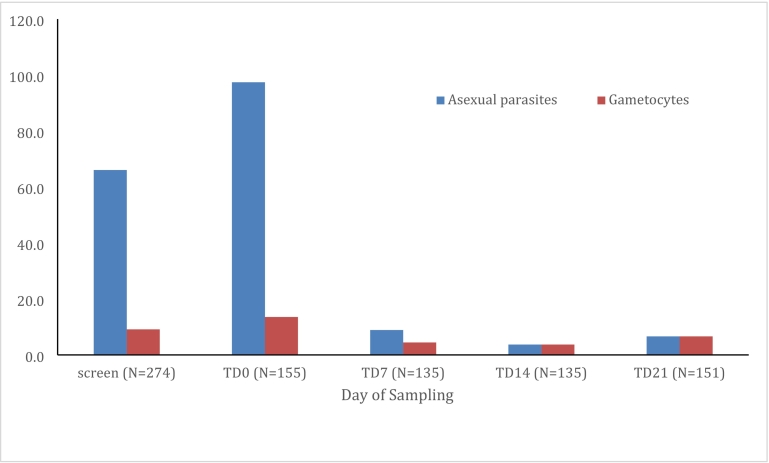
The prevalence of gametocytes and asexual parasites over 5 sampling times. On visit 1, school children were screened to identity parasitaemic individuals. On visit 2 (TD0), a second blood sample was taken and DP administered to children who were microscopically confirmed as parasitaemic on visit 1 (day of screening). These participants were followed up weekly for 3 more weeks. The days of screening and treatment (TD0) are indicated. DP: dihydroartemisinin-piperaquine, screen: day of screening was at visit 1, which is 7 days before treatment, TD0: day of DP treatment, TD7, TD14 and TD21 days of active follow-up.

**Fig. 2:**
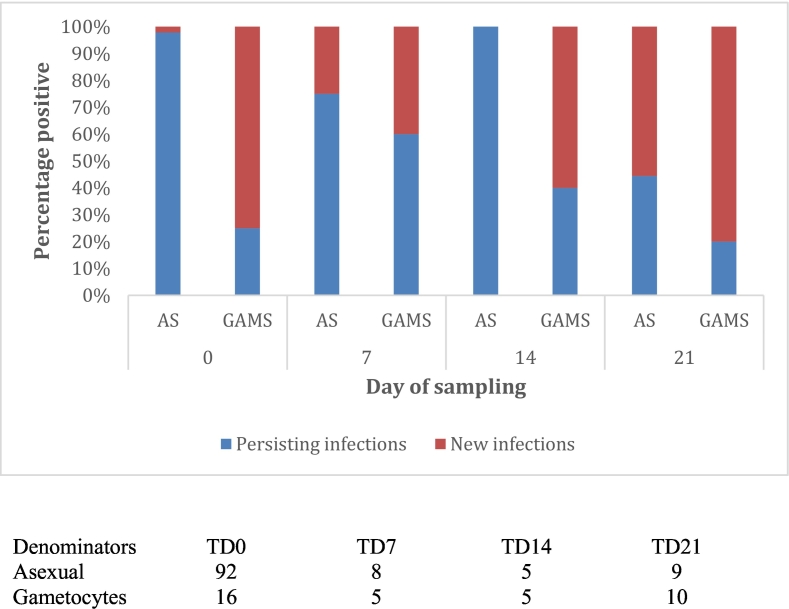
Percentage of children with persisting and new asexual parasites and gametocytes during active follow-up. The percentage of children with asexual parasites and gametocytes from the most recent previous sampling day (persisting asexual parasites and gametocytes) and new parasites (new asexual parasites and gametocytes) are indicated for day of treatment, 0, 7 days after treatment, 14 and 21 days' post-treatment.

Of the 274 children who were screened for malaria parasites by thick and thin blood smears on the day of screening (D-7), 181 (66%) were parasite-positive by microscopy. Of these, 155 remained parasitaemic the following week at visit 2 and received the 3-day treatment with DP (TD0). These were followed up weekly for 3 weeks until visit 5 (TD21). The mean age for enrolled and treated children was 10 years (Table 1) while the male to female ratio was 82:73. Microscopic gametocyte prevalence at visit 1, day of screening, was 9% (25/274). At enrolment, TD0, the microscopic gametocyte prevalence was 13.6% (21/155) (Fig. 1). The geometric mean gametocyte density at visit 1 (screening) was 21.49 (CI: 15.22–30.33) gametocytes per microliter of blood (Table 1) which was not significantly different statistically from geometric mean gametocyte density at TD0 (geometric mean = 16.70 (CI: 15.23–18.32), *p*-value = 0.127 (Table 1). No evidence of significant increase or decrease in gametocyte density was found between visit 1 and visit 2 in a pairwise test of symmetry (McNemar's test; *P* = 0.446). Similarly, there was no significant difference statistically in the geometric mean of asexual parasite densities between the day of screening (geometric mean = 74.69 (CI: 53.16–104.95) and the day of enrolment (geometric mean = 92.48 (CI: 58.65–145.82), *p*-value = 0.283 (Table 1). One hundred and fifty-five children received a full curative treatment course of DP under daily supervision for 3 days. Of the 21 participants with gametocytes on TD0, 19% (4/21) of these had gametocytes 7 days prior at visit 1 (Fig. 1). (See Fig. 2.)

**Table 1:** Baseline characteristics of asymptomatic school children at screening (day −7) and treatment days (day 0).

	Day −7	Day 0	P-value
Number	274^1^	155^1^	
Female: Male	138:136	73:82	
Mean age (95% CI)	10 (9.7–10.3)	10 (9.6–10.5)	
Geometric mean asexual parasite density (95% CI)	74.69 (53.16–104.95)	92.48 (58.65–145.82)^2^	0.283
Geometric mean gametocyte density (95% CI)	21. 4 (15.21–20.33)	16.7 (15.21–18.32)	0.127

1 Two hundred and seventy-four school children were screened for malaria parasites (day −7), of which 155 were parasite positive and also available one week later for DP treatment (day 0) and further follow-up and finger-prick blood sampling.

2 Three individuals on day 0 had only detectable gametocytes by microscopy, hence 152/155.

### 3.2 Gametocyte carriage during following-up

As expected, the prevalence of gametocytes and asexual parasites was reduced after treatment at TD7 and TD14 (Fig. 1). The reductions in the prevalence of gametocytes and asexual parasites were significantly different statistically (data not shown). By TD21 both asexual parasites and gametocytes remained detectable in a minority of participants (Fig. 1). Of these, 10 participants that were gametocyte negative at TD14 harbored gametocytes by microscopy at TD21. These sexual stages were either newly emerged, or below the limit of detection by microscopy at TD14.

Using a binary code to represent the presence and absence of gametocytes, we generated 14 patterns of gametocyte carriage as per our protocol for study participants. Over the 5 weeks, 69% (80/116) of all participants did not carry gametocytes at any point in time. Eleven percent (13/116) (Table 2) of them carried gametocytes at a single time point only, whereas 10% had microscopically detectable gametocytes more than once during the days of follow-up. Two children harbored gametocytes continuously for four of the five weeks they were monitored; one carried gametocytes on D-7, D0, D7 and D14, the other harbored gametocytes continuously from D0 until D21 after treatment. None of the school children carried gametocytes throughout the entire study as detected by microscopy (Table 3).

**Table 2:** Patterns in the carriage, development and loss of gametocytes in 116 children followed over the 5 week sampling period.

Gametocyte code/ day of sample collection visit 1-TD0-TD7-TD14-TD21 ⁎	Number	Percent
00000	80	69
00001	4	3
00010	1	1
01000	8	7
01001	2	2
01100	2	2
01111	1	1
10000	12	10
10100	1	1
11000	1	1
11001	1	1
11010	1	1
11011	1	1
11110	1	1
Total	116	100

⁎ Gametocytaemia is presented as a binary code for each of the 5 sampling times, where 0 denotes absence of gametocytes and 1 denotes its presence. Visit 1: day of screening which is 7 days before treatment, TD0: day of DP treatment, TD7, TD14 and TD21 represent 7, 14 and 21 days after treatment with DP. Only per protocol data, from children who were available at all 5 sampling times, are presented.

**Table 3:** Uni- and multi-variate analysis of risk factors for gametocyte carriage during post-treatment follow-up, after adjustment for pre-treatment asexual parasite density.

Risk factors for gametocyte persistence⁎	N	Univariate OR (95% C.I.)	*P* value	*N* = 110	Multivariate OR (95% C.I.)	P. value
Asexual parasite density at TDO (parasites/mL)	111	1.00 (0.9999–1.000)	0.343		1.00 (1.00–1.00)	0.059
Asexual parasites present at both TD1 and TD7	115	15.23 (1.289–179.9)	**0.031**		28.567 (1.456–560.4)	**0.027**
Gametocytes present at visit 1	118	3.462 (1.021–11.74)	**0.046**		5.173 (0.9353–28.61)	0.060
Gametocyte present at TD0	111	17.89 (4.555–70.25)	**<0.001**		22.67 (4.616–111.4)	**<0.001**

⁎ Defined as having gametocytes on TD7 and/or on any day beyond (see Table 2). Both univariate and multivariate analyses were adjusted for baseline asexual parasite density.

Exploratory univariate analyses were carried out for risk factors that, *a priori,* could potentially be associated with post-treatment gametocyte carriage. Age in years, stated gender, asexual parasite density in peripheral blood films at visit 1 or visit 2 and gametocyte density in peripheral blood films at visit 1 were not associated with gametocyte carriage in follow-up. However, persistent asexual parasites at TD7 (visit 3), pre-treatment gametocyte density at visit 2 (TD0) and presence of gametocytes at either visits 1 or 2 were found to have associations with gametocyte carriage in follow-up, and were deployed together in a multiple logistic regression model to identify which, if any of these inter-related parameters might dominate as a risk factor (Table 3). Of these, persistent asexual parasites at TD7 and gametocyte carriage at TDO were significantly associated with persistence of gametocytaemia after treatment, after correction for asexual parasite density at time of treatment.

## 4 Discussion

Here, we examine potential risk factors for gametocyte carriage in a longitudinal study where gametocytaemia was monitored in a cohort of asymptomatic school children infected with *P. falciparum* and treated with dihydroartemisinin-piperaquine. This is ancillary to a major study of antibody responses to *P. falciparum* gametocytes (Dinko et al., 2016). Microscopically-detectable gametocytes were seen in 31% of the asymptomatic school children at one or more timepoints, and these fluctuated over the 5-week follow-up period. Prior to treatment, gametocytaemia inversely correlated with the age of participants and also strongly associated with lower asexual parasitaemia. Thirteen percent of children carried gametocytes after drug treatment (Table 2). Asexual parasites persisted in a small fraction of individuals after supervised treatment, with 4% of our population still parasite positive on day 14 after initiation of treatment. The carriage of gametocytes prior to treatment and persistence of microscopy-detectable asexual parasite carriage 7 or more days post-treatment were both associated with post-treatment gametocyte carriage.

In this study we found a microscopic gametocyte prevalence of 13.6% among the asymptomatic enrolled population. In addition, there were some newly identified gametocyte carriers after DP treatment. Most antimalarial drugs including piperaquine do kill the earliest developmental stages of gametocytes but the later stages are unaffected (Adjalley et al., 2011). Dihydroartemisinin, the active metabolite of all artemisinins, is able to kill stages I-III of gametocytes *in vitro* and *in vivo* but has incomplete activity against stages IV and V gametocytes (Sutherland et al., 2005; Adjalley et al., 2011; White, 2008). Other studies have observed microscopically detectable gametocytes after DP treatment, and successful infection of *Anopheles* mosquitoes in membrane-feeding assays (Sawa et al., 2013). Our observations suggest that mature gametocytes can persist for up to 3 weeks after DP treatment (not including the one-week period of development while in sequestration); the full period of our follow up. Nine percent of the children (12/135) were found to carry asexual parasites one week after treatment with DP and 50% (6/12) of these were carriers of gametocytes. We did not formally test by genotyping whether parasites detected after treatment were actually due to recrudescence or reinfections due to resource constrains, but given the short follow-up period, and the long prophylactic effect of piperaquine (Adjalley et al., 2011), we assume all recurrent asexual parasites had survived drug treatment.

The persistence of gametocytes after treatment is likely to reflect both persistent and newly-emergent gametocytes following ACT treatment, the latter indicating the presence of some persisting asexual parasites leading to *de novo* gametocyte production. In this context our observation of a positive association between “persisting” asexual parasitaemia and the likelihood of gametocytaemia 1 to 3 weeks after DP treatment is relevant and needs verification in future studies designed to measure drug efficacy.

At least 5% of the children at any sampling time point harbored detectable gametocytes, but estimated densities fluctuated owing either to the low sensitivity of microscopy in detecting gametocytes or to emergence of new gametocytes. Gametocytaemia was found to be inversely correlated with the age of the school children as shown in previous studies (Bousema and Drakeley, 2011; Drakeley et al., 2000; Bousema et al., 2004; Dunyo et al., 2006). We also observed gametocyte carriage to be associated with lower asexual parasite density, although this was not statistically significant, suggesting larger studies with greater power are required to further explore this relationship in the Ghana setting.

Despite observations made in this study, there are several studies showing evidence of efficacious treatment outcomes of DP in symptomatic individuals from different malaria endemicities. In an area of unstable malaria transmission in Sudan, Mohamed et al. (2017) showed that DP is efficacious in the treatment of uncomplicated malaria, where only one parasitological treatment failure was recorded 35 days after dosing as compared to other ACTs (Mohamed et al., 2017). Clinical trials in other African countries such as Kenya, Angola, Ghana, Cameroun and Niger have also shown similar efficacies for DP in the treatment of uncomplicated malaria in children (Ogutu et al., 2014; Plucinski et al., 2015; Adjei et al., 2016; Nji et al., 2015; Grandesso et al., 2018 and Davlantes et al., 2018), though gametocytaemia was not evaluated in most studies. A few studies exist where DP cleared all asexual parasitaemia among asymptomatic individuals before day 3 (Okebe et al., 2016) and day 7 (Dicko et al., 2018).

It should be noted that our study was not designed as a clinical trial and, being conducted in asymptomatic children, there was no drug efficacy end point. Despite the above caveat, it is still unclear why our study demonstrates markedly different clearance kinetics of asexual parasites, and suggests that DP is less effective in the absence of a high-level anti-parasitic immune response as evidenced by the presence of malaria symptoms. Other possible explanations for our findings include poor drug absorption or sub-standard medicines. We have evidence to rule out the latter as tests of drug quality revealed that the batches of DP used in the study met the required standards (Dinko et al., 2013).

The primary limitation of this study is our reliance on standard microscopy for gametocyte detection, which is known to underestimate gametocytaemia (Dinko et al., 2013). Detecting gametocytes by RNA-based methods such as nucleic acid sequence-based amplifications (NASBA) or RT-qPCR are likely to have detected a higher prevalence of gametocyte carriage (Schneider et al., 2004; Dinko et al., 2018). Future studies should employ molecular methods in the detection and quantification of gametocytes *in vivo*.

## 5 Conclusions

In this study we showed that microscopically detected gametocytes are abundant in school children and persistently fluctuated between sampling time points. Asexual parasites also persisted in a minority of treated individuals. These data imply that asymptomatic children in populations receiving MDA with DP could be an important reservoir of both persisting asexual parasitaemia and post-intervention transmission. Further studies deploying molecular characterization of gametocytes in asymptomatic individuals are required to understand why gametocytes and asexual parasites persist after DP treatment. These findings will have important implications for successful deployment of DP in community treatment campaigns to reduce malaria transmission.

## Author contributions

BD and CJS conceived and designed the study. DA, NKB and JAL contributed to fieldwork. BD performed the experiments and analysed the data with TB and CJS. BD wrote the first draft of the paper. All authors read and approved the final manuscript.

## Availability of data and materials

The data used and or analysed during the study are available from the corresponding author on reasonable request.

## Ethics approval and consent to participate

The study was approved by the Ghana Health Service Ethics Committee and the Ethics Committee of the London School of Hygiene and Tropical Medicine. Informed consent was obtained from all individuals included in this study.

## Funding

Bismarck Dinko was funded by a PhD Fellowship from the 10.13039/501100003419Ghana Education Trust Fund. The research and field studies were funded by the 10.13039/100009660London School of Hygiene and Tropical Medicine, UK.

## Declaration of Competing Interest

The authors declare that there are no competing interests.

## Appendix A Supplementary data

Supplementary material Detection of persistent and new asexual parasitaemia and gametocytaemia over 5 weekly follow-up period. Image 1
