# Supplementary material for: Prevalence of Plasmodium falciparum gametocytaemia in asymptomatic school children before and after treatment with dihydroartemisinin-piperaquine (DP)
Source: Parasite Epidemiol Control. 2023 Feb 17;21:e00292. doi: 10.1016/j.parepi.2023.e00292 (PMC9969054; doi:10.1016/j.parepi.2023.e00292)
Supplement: Supplementary file 1 — Supplementary material Detection of persistent and new asexual parasitaemia and gametocytaemia over 5 weekly follow-up period. [file mmc1.docx]

**Supplemental table 1: Detection of persistent and new asexual parasitaemia and gametocytaemia over 5 weekly follow-ups.** Persistent parasitaemia represent parasites detected between different follow-up days and there was no genotyping to substantiate the fact these were the same parasites but they come from the same individuals. New parasitaemia represent parasites found in individuals that were parasite negative the previous follow up. D-7: day of screening for parasitaemic children which is 7 days before treatment, TD0: day of DP treatment, TD7, TD14 and TD21 represent 7, 14 and 21 days after treatment with DP respectively. In this case sub-total asexual parasitaemia and gametocytaemia does not include the number of individuals who were positive and later lost their parasitaemia or gametocytaemia.

| Sampling day | Number of participants | Persistent asexual parasitaemia | New Asexual parasitaemia | **Sub-total number parasitaemic** | Persistant gametocytaemia | New gametocytaemia | **Sub-total number gametocytaemic** |
| --- | --- | --- | --- | --- | --- | --- | --- |
| -7 | 274 |  |  |  |  |  |  |
| TD0 | 155 | 90 | 2 | **92** | 4 | 12 | **16** |
| TD7 | 135 | 6 | 2 | **8** | 3 | 2 | **5** |
| TD14 | 135 | 5 | 0 | **5** | 2 | 3 | **5** |
| TD21 | 151 | 4 | 5 | **9** | 2 | 8 | **10** |
| **Total** | **849** | **105** | **9** | **114** | **11** | **25** | **36** |
